# Postpartum deep vein thrombosis and pulmonary embolism in twin pregnancy: undertaking of clinical symptoms leading to massive complications

**DOI:** 10.1186/1477-9560-11-4

**Published:** 2013-02-22

**Authors:** Leslie Fiengo, Federico Bucci, Gregorio Patrizi, Domenico Giannotti, Adriano Redler

**Affiliations:** 1 Department of Vascular Surgery, La Sapienza University of Rome, Rome, Italy; 2 Vascular Surgery Department, Centre Hospitalier Sud Gironde, Langon, France; 3 Dipartimento Scienze Chirurgiche-Università La Sapienza, Viale Regina Elena, 324-00161, Rome, Italy

**Keywords:** Twin delivery, Postpartum ovarian vein thrombosis, Pulmonary emboli

## Abstract

**Background:**

Deep Vein Thrombosis (DVT) is an important cause of morbidity and is the first cause of maternal death after delivery in Western Nations. The risk of venous thromboembolism is present throughout the pregnancy and is maximal during postpartum, especially after twin delivery. Many of the signs and symptoms of DVT overlap those of a normal pregnancy causing difficulty for diagnosis.

**Case report:**

We report the case of a 33 year-old woman transferred to our Department one week after caesarean section for twin delivery. She presented with severe abdominal pain, fever, abdominal distension and shortness of breath. She had no personal or family history of thromboembolism. Computerized Tomography Scan revealed right ovarian vein thrombosis, left renal vein thrombosis extending up to the Inferior Vena Cava and pulmonary embolism with bilateral pleural effusion. Caval filter was positioned and anticoagulation therapy associated with antibiotics was instituted. Pancreatitis showed up two days after and was promptly treated. Three months after discharge the caval filter was removed and oral anticoagulation was stopped. During a 12-months follow-up, she remained stable and symptom free.

**Results:**

Ovarian vein thrombosis is rare but recognition of signs and symptoms is fundamental to start adequate therapy and avoid potential serious sequelae. The risk for maternal postpartum ovarian vein thrombosis is increased by caesarean section delivery of twins. Such patients should be closely monitored. We illustrated how an underestimated condition can lead to massive complications.

## Introduction

During the third trimester and especially in the first two weeks following delivery the risk of Venous Thromboembolism (VTE) increases in women and it is the leading cause of maternal death in Western Countries [1,2]. VTE risk is five times greater during the postpartum period than during pregnancy [3] and postpartum ovarian vein thrombosis (POVT) is an uncommon complication in pregnancy with a prevalence of 0.15-0.18% [4].

POVT is the result of bacterial infections, hypercoagulability and reduced blood flow in dilated ovarian veins [5,6]. Eighty to 90% occur in the right side due to the dextrotorsion of enlarging uterus with compression of right ovarian vein.

Symptoms include fever, abdominal pain, nausea, vomiting. Complications of POVT are rare [5,7] and can involve renal vein, Inferior Vena Cava (IVC) and lead to pulmonary embolism (PE) which is a life-threatening condition and has been reported in 13.2% of patients with POVT [8].

## Case report

A 33-year-old woman gave birth to twins in March 2011 with caesarean section delivery. She was at her second pregnancy and had no family history of VTE or obesity. The delivery occurred without complication, with an Apgar score for each baby of 10. Two days later, the patient presented symptoms of high fever (up to 42°C) with chills. Her blood pressure was 90/50 mmHg and her pulse was 110 b.p.m.. Clinical examination revealed right lower quadrant pain with rebound tenderness. The patient stated that at 22 week of pregnancy, she referred to her doctor right lower abdominal pain but no further investigations were performed at that time. Since there was no possibility to perform a colour Doppler ultrasound or an ultrasound examination of the Abdomen, she was transferred one week later in our Department with the only laboratory data at hand being: hemoglobin 8 g/dL (range 12–15 g/dL); lipase 128 UI/L (range 13–60); amylase 183 UI/L range (20–100); platelets 150,000 ((150–450) x 10^9^/L); PT 5.06 (0,8-1,2); PTT ratio 2.21 (0,8-1,2); fibrinogen 866 mg/dL (200–400); AT III 81%(80–120); D-Dimer 3376 μg/L (range 0–550).

**Figure 1:**
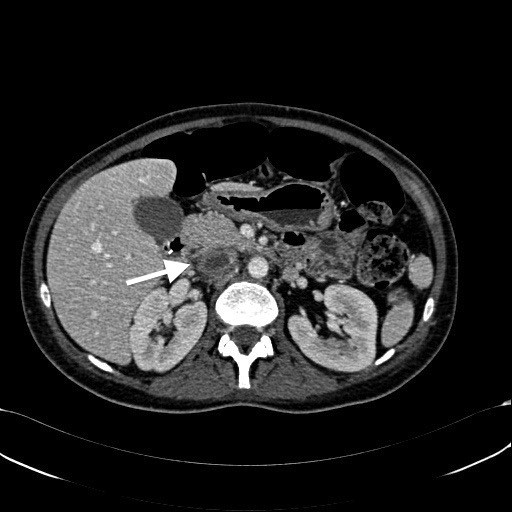
Abdominal Computed Tomography showing complete thrombosis of Inferior Vena Cava.

**Figure 2:**
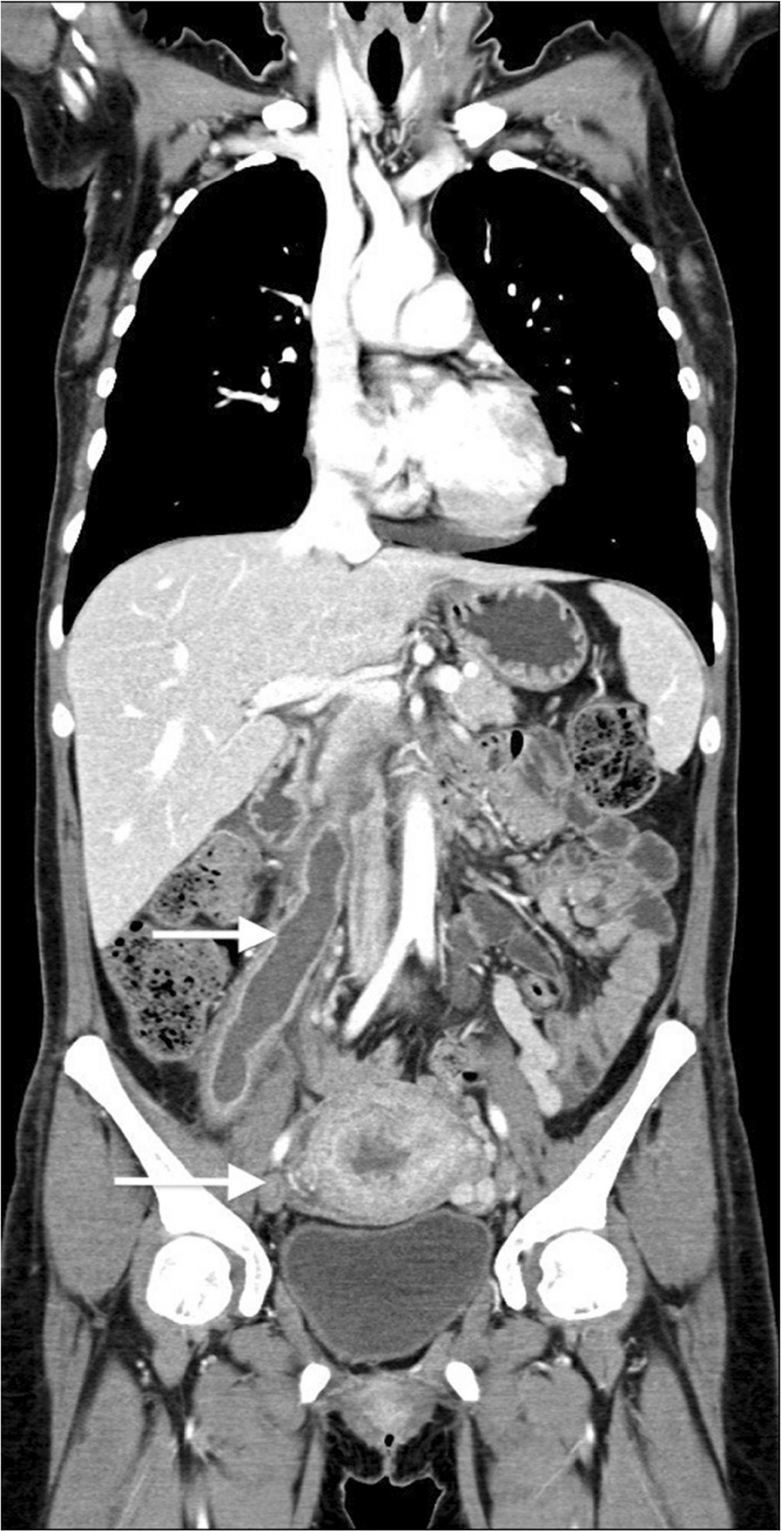
CT-scan showing complete thrombosis of IVC, right ovarian vein and pelvic varicosity.

Computed Tomography (CT) of the abdomen revealed right ovarian vein thrombosis (ROVT) with a diameter of 28 mm and thrombosed left renal vein (LRV) with a maximal diameter of 18 mm, therefore the patient was treated with intravenous Heparin for 5 days (25.000UI/24 at 20 mL/h) and antibiotics. Six days after she complained difficulty of breath and thoracic pain. CT-scan of the thorax revealed PE with bilateral pleural effusion, diffuse pelvic varicosity with complete ROVT (diameter of 44 mm) extending to the IVC for 3 cm leading to an almost complete obstruction right up to under the renal veins with partial obstruction of the Iliac bifurcation and External Iliac Vein bilaterally (Figure 1 and 2). Caval filter (ALN) was placed under local anaesthesia below the renal veins with a right jugular puncture.

Anticardiolipin, antiphospholipid antibodies, Protein C and S, antithrombin III (100;range 80–120), Factor V Leiden were assessed to rule out congenital thrombophilia. Intravenous heparin was suspended and low molecular weight heparin (Clexane®) was started. Since the patient referred abdominal pain in the upper left quadrant irradiated to the back, laboratory tests were performed showing amylase 182 UI/L (range 20–100) and lipase 204 (range 13–60) as initial pancreatitis. Abdominal ultrasound demonstrated the presence of gallbladder stones, one of the multiple cause of pancreatitis in pregnant women. For this reason the patient underwent total parenteral nutrition (TPN) and standard medical treatment. After one month the patient fully recovered and was discharged with anticoagulant treatment (Coumadin®, maintaining an INR between 2.0 and 3.0). Caval filter was removed three months later, after a control CT-scan that showed the reduction of the ROVT, LRV and IVC thrombus. The patient is stable and symptom free at present.

## Discussion

VTE remains the most common cause of direct maternal deaths [9]. POVT can occur after delivery causing severe complications. In pregnancy there is a progressive alteration in the balance between prothrombotic and anticoagulant factors, which both increase fibrin deposition and reduce fibrinolysis, resulting in a procoagulant state [10]. Moreover, flow velocity in the lower limbs is reduced by approximately 50% by the third trimester [11] and 50% of cases of VTE in pregnancy are associated with inherited or acquired thrombophilia [12]. In ninety percent of the cases, the right ovarian vein is involved due to the incompetence of the valves [12]. Enlarged pregnant uterus in twins may cause obstruction of IVC leading to vein stasis [13] and its complications. Salomon and Dutizky [5,7] wrote the first report of an association with twin delivery and the risk of POVT. The Author concluded that the size of the uterus during twin pregnancy resulted in compression of the ovarian vein. However all of their patients affected underwent artificial reproductive techniques in order to become pregnant, which was not the case of our patient. The most important risk factors are multiparity, puerperium, post-operative periods, infections, neoplasm, systemic lupus erythematosus and hypercoagulability states [1]. Typical presentations include abdominal pain, pelvic or buttock pain in iliac DVT and neck pain when associated with Internal Jugular Vein thrombosis. Clinical presentation includes fever and pain to the right iliac fossa. Differential diagnostic with appendicitis, sepsis, ovarian torsion is mandatory and must be done in early stages to avoid massive thrombosis and PE, which can be fatal in some cases. Unusual presentation includes Budd-Chiari syndrome and cerebral vein thrombosis (CVT). In the study of Salomon and Dulitzky, only in 23% of the cases thrombophilia was discerned. Salomon et al [5]. concluded that the risk for maternal POVT is increased by caesarean delivery of twins.

Early diagnosis may be done with compression ultrasonography (CUS) that has a sensitivity of 97-100% and a specificity of 98-99% [14], while contrast-enhanced CT and Magnetic Resonance Imaging (MRI) may confirm the diagnosis and quantify thrombosis extension and PE which occurs in 13% of cases [15]. A thick-walled, enlarged ovarian vein with rim enhancement and central hypodensity are considered the main CT findings in POVT [16]. If abdominal pain is present ultrasounds should be routinely performed during pregnancy to exclude ovarian thrombosis. Recent studies suggested that the risk of POVT increase after caesarean section [1]. Broad-spectrum antibiotic treatment should be settled immediately, as should intravenous heparin or low molecular weight heparin (LMWH); once thrombolysis has begun, oral anticoagulants must be introduced and continued for 3 to 6 months. Despite the use of LMWH, DVT and embolism may develop [17]. The optimum length of time for maintaining anticoagulation in these patients is unknown (5), therefore we may consider the possibility to continue therapy at least for 3 months with laboratory control.

Caval filter is recommended in extensive DVT and whenever discontinuation of anticoagulation might carry high risk of PE [4]. Placement of VCF are particularly considered in case of a high bleeding risk, or other contraindications, precluding the use of therapeutic doses of Anticoagulation [1,4]. There are no studies regarding the use of graduated elastic compression stockings (GCS) in pregnant women. However it is likely that stockings could be beneficial in this scenario [18]. A meta-analysis in non-pregnant patients showed a reduction in risk of Post-thrombotic syndrome (PTS) and severe PTS in patients using GCS after diagnosis of thrombosis.

Hence the recommendation from the Royal College of Obstetricians and Gynaecologists is that GCS (knee-length with compression strength of 30–40 mm Hg) should be applied to help prevent PTS [18].

This leads to the recommendation that all women should be assessed for the risk factors of VTE in early pregnancy and that the assessment should be repeated if the woman is admitted to hospital or develops intercurrent problems. The assessment should be repeated anyway intrapartum or immediately postpartum [18].

In our reported case the complications were not prevented since the diagnosis and the therapy were performed too late. Maybe complications could have been avoided if early ultrasound was performed during pregnancy as soon as the patient was complaining of groin pain with no relief.

A better recognition of POVT syndromes leads to earlier diagnosis and more favorable clinical outcomes.

## Consent

Written informed consent was obtained from the patient for publication of this Case report and any accompanying images. A copy of the written consent is available for review by the Editor-in-Chief of this journal.

## Competing interest

All authors declare that they have no competing interest.

## Authors’ contribution

FL and BF drafting the manuscript and bibliographic research and interventional procedure. GD,PG drafting the manuscript, revision of manuscript and acquisition of data of manuscript and bibliographic research. RA-Chief of the Department, final approval of the manuscript. All authors read and approved the final manuscript.
